# The Chitinases as Biomarkers for Amyotrophic Lateral Sclerosis: Signals From the CNS and Beyond

**DOI:** 10.3389/fneur.2020.00377

**Published:** 2020-05-27

**Authors:** Nayana Gaur, Caroline Perner, Otto W. Witte, Julian Grosskreutz

**Affiliations:** ^1^Hans Berger Department of Neurology, Jena University Hospital, Jena, Germany; ^2^Center for Immunology and Inflammatory Diseases, Massachusetts General Hospital, Charlestown, MA, United States; ^3^Jena Center for Healthy Ageing, Jena University Hospital, Jena, Germany

**Keywords:** neurodegeneration, biomarker (BM), neuroinflammation, chitinases, amyotrophic lateral sclerosis (ALS)

## Abstract

Amyotrophic lateral sclerosis (ALS) is a late-onset neurodegenerative condition, most widely characterized by the selective vulnerability of motor neurons and the poor life expectancy of afflicted patients. Limited disease-modifying therapies currently exist, which only further attests to the substantial heterogeneity associated with this disease. In addition to established prognostic factors like genetic background, site of onset, and age at onset, wide consensus on the role of neuroinflammation as a disease exacerbator and driver has been established. In lieu of this, the emerging literature on chitinases in ALS is particularly intriguing. Individual groups have reported substantially elevated chitotriosidase (CHIT1), chitinase-3-like-1 (CHI3L1), and chitinase-3-like-2 (CHI3L2) levels in the cerebrospinal, motor cortex, and spinal cord of ALS patients with multiple—and often conflicting—lines of evidence hinting at possible links to disease severity and progression. This mini-review, while not exhaustive, will aim to discuss current evidence on the involvement of key chitinases in ALS within the wider framework of other neurodegenerative conditions. Implications for understanding disease etiology, developing immunomodulatory therapies and biomarkers, and other translational opportunities will be considered.

## Introduction

### ALS and Neuroinflammation

Amyotrophic lateral sclerosis (ALS) is the most prevalent form of adult-onset motor neuron disease and clinically presents with the relentless destruction of primarily (but not exclusively) upper and lower motor neurons (UMN, LMN). Riluzole, the sole treatment available, confers only modest effects via a median increase of 2–3 months in survival; most patients eventually succumb to respiratory failure. Although there is a pressing need for treatment modalities that tackle disease aggressiveness, therapeutic development has been severely constrained by the disease's characteristic heterogeneity; this stems from age-at-onset and site-of-onset, presence of disease-associated mutations, and comorbidities, including frontotemporal dementia (FTD) (1). Progression and survival rates are also highly variable; while the median survival is 2–3 years from symptom onset, some patients present with a disease duration of over 10 years (2). Cellular and animal studies have provided elegant evidence that neuroinflammation contributes to ALS pathology and that concomitant glial dysregulation is necessary for motor neuronal degeneration (3–5). Numerous immunological changes, including the functional alteration and pro-inflammatory phenotype of circulating myeloid cells (6), dysregulated leukocytic chemokine receptor expression (7), the reduction of regulatory T cells (8), and cytotoxic T cell infiltration, have also been reported in patients (9).

Despite this, there remains a paucity of biological tools that adequately capture the neuroinflammatory response across the disease; this may partially explain the failure of immunomodulatory therapies to date. Biomarkers that reflect target engagement and assess the efficacy of novel treatments are therefore crucial. Although molecular imaging studies of microglial activation are underway, fluid-based biomarkers are more accessible and can provide important insights into disease pathomechanisms. For instance, cerebrospinal fluid (CSF) and humoral levels of the neurofilament proteins have been validated as robust diagnostic and prognostic markers for ALS. Several inflammatory cytokines have also been reported as dysregulated in ALS, including TNF-α, MCP-1, and IL-6 (10–12). In lieu of this, recent reports of elevated chitinase levels in ALS are particularly interesting, as these have already been reported as surrogate markers of a chronic inflammatory response in non-neuronal conditions.

### Mammalian Chitinases: Novel Players in Neurodegeneration?

The chitinases belong to the family 18 glycosyl hydrolases (GH18) and are characterized by their ability to cleave chitin, a natural polysaccharide found in the coating of various pathogens. The GH18 family is ubiquitously expressed across a wide range of organisms, from bacteria to humans; evolutionary conservation in the latter is particularly interesting, given the lack of endogenous chitin synthesis. This has led to the view that chitin is a defense target for the mammalian immune system or an “immune stimulator.” Indeed, it is recognized by several pattern recognition receptors and can trigger associated immune responses in a fragment-size and tissue-dependent manner (13). Mammalian chitinases include the enzymatically active chitinases chitotriosidase (CHIT1) and acidic mammalian chitinase (AMCase) that can degrade chitin, and the chi-lectins (CLs) chitinase 3-like 1 and -like 2 (CHI3L1, CHI3L2). Despite being able to bind chitin with high affinity, the CLs possess no chitinolytic activity, owing to the absence of the catalytic motif. CHIT1 is primarily expressed by cells of myeloid lineage, particularly mature macrophages (14, 15). Like CHIT1, CHI3L1 is absent in monocytes and strongly upregulated during later stages of macrophage differentiation (16). CHI3L1 is also produced by reactive astrocytes and associated with chronic neuroinflammation, as will be further discussed in the Section Chitinases Across the ALS-FTD Spectrum (17–19). While CHI3L2 hasn't been as extensively studied, expression has been noted in chondrocytes, synoviocytes, and alternatively activated “M2” macrophages (20).

**Figure 1:**
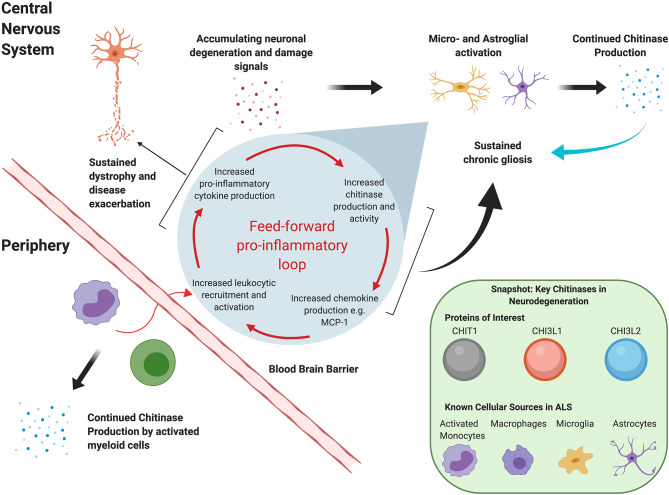
Hypothetical mutual regulation cascade of chitinases and the mammalian immune system that sustains neuroinflammation in ALS.

Although the exact roles of these moieties remain to be fully elucidated, it is clear that they extend beyond innate immunity against chitin-containing pathogens. Chitinases have been reported in the context of adaptive Th2 response mediation (21, 22), tissue remodeling and repair, and, most recently, oligodendrogenesis (23). Dysregulated chitinase levels have been reported in several chronic neurodegenerative conditions, including Alzheimer's disease (AD) and FTD. *In vitro* evidence suggests that, at least in ALS, they may act in a “feed-forward” loop that sustains neuroinflammation and exacerbates disease, as illustrated in Figure 1. For instance, in a transgenic rat model, TDP-43 induced astrocytic *CHI3L1* up-regulation; in turn, synthetic CHI3L1 caused neuronal death in a dose-dependent manner (19). Similarly, Raju et al. reported that CSF from ALS patients impacted cell viability and upregulated CHIT1 expression in murine microglial cultures (24). Subsequent exposure to CHIT1 itself caused microglial activation, indicating again a “self-propagating” inflammatory mechanism (25).

This review, while not exhaustive, will summarize current evidence for chitinase dysregulation in ALS and its implications for understanding disease etiology and progression, and therapeutic and biomarker development. CHIT1, CHI3L1, and CHI3L2 will be focused on, since these have been most extensively studied in a neurodegenerative context.

## The Chitinases in ALS

### Evidence Concerning Diagnostic Potential

Varghese et al. (26) were the first to report chitinases in the context of ALS; using quantitative mass spectrometry (MS) and ELISA-based validation in an independent cohort, they showed that CSF levels of CHIT1, CHI3L1, and CHI3L2 were significantly elevated in ALS patients relative to healthy controls (HCs). This elevation has since been confirmed by several studies using a range of proteomic and transcriptomic methods (27–33). Recent studies have predominantly focused on assessing discriminatory power with regard to mimic conditions, and other neurodegenerative diseases. However, studies have differed with respect to (a) the chitinases and secondary targets investigated; (b) cohort demographics; (c) bio-fluids assessed, and (d) experimental and analytical methods used (Table 1). Thompson et al. subsequently investigated all three chitinases and reported that they were significantly higher in the CSF of ALS patients relative to HCs, mimics, and asymptomatic mutation carriers (MCs) and that increased CHIT1 levels corresponded to active forms of the enzyme. While all three could reliably distinguish ALS from HCs and mimics, they were outperformed by pNfH. Furthermore, all three chitinases performed poorly in distinguishing ALS from primary lateral sclerosis (PLS) (28). A prior MS-based study by the same group also noted a modest fold change for only CSF CHIT1 and CHI3L2 between ALS and PLS (31). Similarly, while Steinacker et al. (33) recommended CHIT1 as a potential differential diagnostic marker for ALS, they also noted that levels were increased in other neurodegenerative conditions and that pNfH and NfL had superior discriminatory power. In the same vein, Gille et al. (10) reported that elevated CSF CHIT1 and CHI3L1 levels were only weakly specific to ALS patients relative to neurological disease controls (NDCs). Observations of significant ALS-associated chitinase elevations in blood have been limited, barring one study that reported significantly elevated CHIT1 activity in dried blood spots (30) and another that noted higher CHIT1 levels in a genetic ALS (gALS) cohort (27) (both relative to HCs). This, coupled with reports of poor correlations between peripheral and CSF chitinase levels, makes a blood-based marker unlikely.

**Table 1:** An overview of recent studies investigating chitinases in the context of ALS.

**Study PMID**	**Study targets**	**Participants**				**Sample type**	**Methods used**	Relative expression in ALS^*^	Correlation with neurofilament levels^*^	**Other targets**	**Proposed bio mechanism**	**Proposed utility**
		**ALS**	**HCs**	**Mimics NDegs, NDCs**	**Other groups**							
31175169	CHIT1 and CHI3L1	105		16 mimics, 102 NDCs		CSF and serum	ELISA and chemiluminescent assays	CSF CHIT and CHI3L1 ↑^NDCs, Mimics^	All with pNfH and NfL (in CSF)	MCP-1	Surrogate markers of Gliosis	Monitoring therapy response and stratification
31123140	CHIT1, CHI3L1, CHI3L2	82	25	12 mimics, 10 PLS	5 asym gALS MCs	CSF and serum	ELISA, enzymatic activity assay	CSF All ↑^HCs, Mimics, MCs^, CSF CHIT, CHI3L2↑^PLS^	All with pNfH (in CSF)	C-RP	Glial activity	Adjunctive predictor of progression, monitoring glial response to therapy
30224549	CHIT1 CHI3L1	70 sALS, 65 gALS	36 HCs	26 sFTD, 23 gFTD	26 asym. gALS MCs	CSF and blood	ELISA	CSF CHIT↑^HCs, MCs, gFTD^, Blood CHIT↑^HCs^, CSF CHI3L1 for gALS, and gFTD↑^HCs, MCs^	All with pNfH and NfL (in CSF)	GFAP	Microglial activity and astrogliosis. Neuroinflammation is common to gALS and sALS	
30215603	CHI3L1	IHC 11, ELISA 86, RT-qPCR 12 all sALS	IHC 23, ELISA 21, RT-qPCR 10			CSF, blood, spinal cord and frontal cortex PMT	RT-qPCR, IHC, ELISA, IB	PMT *CHI3L1*, CHI3L1 ↑^HCs^, CSF CHI3L1↑^HCs^	NfL	*AIF1, CD68*, IBA1, GFAP	Increased astroglial activity	Potential prognostic marker when used with NfL
29142138	CHIT1	ELISA 60, IHC 3	ELISA 25, IHC 2	ELISA 46 NDCs and 135 Ndegs, IHC NDegs 4		CSF, blood, spinal cord PMT	ELISA, IHC	CSF CHIT↑^HCs^, ^NDCs, NDegs^, Spinal cord PMT CHIT↑^NDegs, HCs^	pNfH and NfL (in CSF)	IBA1, CD68	Microglial/macrophage activation	Biomarker for immune activation; can be used to monitor efficacy of immunomodulatory therapies
29331073	CHIT1, CHI3L1, CHI3L2	43	25	6 PLS, 12 mimics, 20 NDeg		CSF	High throughput MS, ELISA (for pNfH)	CHIT1, CHI3L2 ↑^HCs, Mimics, NDeg, PLS^, CHI3L1↑^HCs, Mimics, NDeg^	All with pNfH		Microglial activity	Distinguishing between ALS and ALS mimic conditions
30134252	CHIT1	29	36			CSF and blood	ELISA, chemiluminescence, enzymatic activity assay	CSF CHIT1 activity↑^HCs^		CCL18, TNF-α, IL6, *CHIT1*	Microglial activation	Neuroinflammation biomarker
30291183	CHI3L1	38	49	86 FTD		CSF	ELISA	CHI3L1↑^HCs^	NfL	sAPPβ		Staging and prognosis along ALS-FTD spectrum
25563799	CHIT1	75	106			Blood	Enzymatic activity assay	CHIT1 activity↑^HCs^		Lysosomal enzyme activity	Microglial activity and possible chronic triggering of the innate immune system	
27634542	CHIT1	40 sALS		40 NDCs		CSF	ELISA	CHIT1↑^NDCs^	pNfH	S100-β, Cystatin C		Improves diagnostic potential when used with pNfH
24295388	CHIT1, CHI3L1, CHI3L2	26 sALS	23			CSF	LC-MS/MS, ELISA, enzymatic activity assay	CHIT1, CHI3L2↑^HCs^, CHIT activity ↑^HCs^		Osteopontin	Microglial activity and potential response to deposition of chitin-like substances in CNS aggregates	

* *Reported results were statistically significant*.

*AIF-1, Allograft inflammatory factor 1; asym, asymptomatic; CCL-18, Chemokine ligand 18; C-RP, C Reactive Protein; CSF, cerebrospinal fluid; ELISA, Enzyme-linked Immunosorbent assay; FTD, frontotemporal dementia; gALS, genetic Amyotrophic Lateral Sclerosis; GFAP, glial fibrillary acidic protein; gFTD, genetic Frontotemporal Dementia; HCs, Healthy Controls; IB, Immunoblotting; IHC, Immunohistochemistry; IL-6, interleukin 6; LC-MS, Liquid Chromatography Mass Spectrometry; MCs, Mutation Carriers; MCP-1, Monocyte chemoattractant protein 1; MS, Mass Spectrometry; NDCs, Neurological Disease Controls; NDegs, Neurodegenerative Disease Controls; NfL, neurofilament light chain; PLS, Primary Lateral Sclerosis; PMT, Post-mortem Tissue; pNfH, phosphorylated neurofilament heavy chain; RT-qPCR, Real-time Quantitative Polymerase Chain Reaction; sALS, sporadic ALS; sAPPβ, soluble amyloid precursor protein beta; sFTD, sporadic Frontotemporal Dementia; TNF-α, tumor necrosis factor alpha*.

Applicability as stand-alone diagnostic markers is also likely to be constrained by the effect of functional variants. For instance, polymorphisms in the *CHI3L1* locus contribute to almost 15% of the variance in CSF CHI3L1 levels (34). Likewise, duplication in exon 10 of the *CHIT1* gene reduces both expression and activity; although this polymorphism is highly prevalent in European populations, no significant differences in genotype frequency have been observed between ALS patients and healthy individuals (27, 30). Additionally, presence of the *CHIT1* polymorphism has no influence on neurofilament levels or age of onset in patients, making a causative role in ALS pathogenesis unlikely. Importantly however, both CHIT1 expression and activity are significantly elevated in ALS patients (relative to HCs) independent of genotype and other factors like gender and age, indicating that disease status—rather than the presence of the polymorphism—determines the extent of dysregulation (12, 27, 30).

### Evidence Concerning Prognostic Potential

The prognostic potential of the chitinases has been examined in relation to several clinical outcomes, including disease severity (overall ALSFRS-R score), the ALSFRS-R-derived progression rate (PR), survival, and disease duration, with several conflicting results as discussed below. The majority of the results discussed here focus on CSF, as almost no robust and consistent links between blood chitinase levels and prognostic factors have been reported. It is worth noting, however, that studies have only now begun to examine CHIT1 enzymatic activity in addition to protein levels and that links between the periphery and prognostic factors, as reported by Pagliardini et al. (30), may yet emerge.

#### Links With Disease Severity and Progression

Evidence for a link with disease severity and progression has been tenuous at best. Martinez-Merino et al. (12) controlled for *CHIT1* genotype and reported that while ALS patients had significantly elevated CHIT1 activity, it correlated with neither disease severity nor progression. Thompson et al. (28) reported a significant albeit modest correlation between CHIT and CHI3L2 levels—but not CHI3L1—and PR after controlling for gender, age at onset, and site of onset; however, a stronger correlation was noted for pNfH. Conversely, Illán-Gala et al. (32) and Andres-Benito et al. (35) reported that CSF CHI3L1 levels correlated with PR to almost the same degree as CSF NfL levels.

Gille et al. (10) noted that both CSF CHIT1 and CHI3L1 only weakly correlated with PR at time of sampling; however, “fast” progressors had significantly higher levels of CHIT1 and CHI3L1 than “slow” progressors. One study reported that CSF CHIT1 also significantly correlated with both disease severity and PR (inversely) and to almost the same magnitude as NfL and pNfH. However, these correlations did not persist when patients were stratified based on PR, despite “fast” progressors having significantly higher levels of CHIT1 (33). Chen et al. (29) too reported no significant differences in CHIT1 levels between PR-stratified patients.

It is worth noting that establishing any association between the chitinases and PR is likely confounded by the lack of any external consensus on the thresholds for “high” or “low” PR. These are often arbitrarily set based on individual cohorts, thus constraining inter-study comparability and potentially occluding genuine biological signals.

#### Links With Disease Duration

Evidence for an association with disease duration has also been inconsistent, even by the few studies that have included longitudinal sampling. CSF CHIT1 activity did not significantly differ between patients stratified based on time since onset to sampling (12). A MS-based study reported a small increase in CSF CHI3L1 levels over time in patients who had low levels at onset (31). However, a subsequent ELISA-based verification noted that CSF chitinase levels in ALS and PLS patients did not significantly increase over a follow-up period of ~2 years, even when patients were stratified by PR (28). Similarly, no significant associations between CSF CHIT1 and CHI3L1 and disease duration were observed in a cohort of 105 ALS patients (10). Indeed, evidence from asymptomatic ALS and FTD MCs suggests that chitinase elevation is a feature of the early symptomatic phase of the disease and is unlikely by itself to trigger disease onset, given that no significant differences were observed between patients with either genetic or sporadic disease (27).

#### Links With Survival and Mortality

Studies examining survival have also reported discrepant results. Di Rosa et al. analyzed microarray datasets and reported that patients with a shorter survival had significantly higher *CHI3L1* and *CHI3L2* in their motor cortex than those that survived longer; levels also inversely correlated with survival in the entire patient cohort (36). Cox proportional hazards analyses have also revealed a significant association between CSF CHIT1 levels and mortality, while one study reported that the association was independent of pNfH levels, another by the same group reported the opposite (28, 31). However, neither study had data on other prognostic factors, e.g., respiratory and *C9orf72* status, thus precluding a definitive conclusion on the influence of CHIT1. In contradiction, Gille et al. (10) reported that CSF CHI3L1, but not CHIT1, significantly affected mortality; this is compelling because they included data for eight established prognostic markers. The authors did not however compare how the chitinases performed relative to neurofilaments. Building on this, Illán-Gala et al. (32) also reported that increased CSF CHI3L1 levels were associated with shortened survival, even after adjustment for sex, age at onset and site of onset, NfL levels, and ALSFRS-R score at time of sampling. Taken together however, the currently available evidence doesn't unequivocally establish the degree to which the chitinases influence survival and whether they outperform established prognostic factors.

#### Links With Additional Indices

Although data are limited, some studies have also begun to examine a wider range of clinical outcomes; for instance, peripheral CHIT1 activity was significantly inversely correlated with forced vital capacity (30). Additionally, CSF CHIT1 and CHI3L1 levels correlated with the number of regions clinically affected by both UMN and LMN and only UMN degeneration, respectively (10, 28). Frontotemporal cortical thickness, as assessed by structural MRI, directly correlated with the CSF sAPPβ:CHI3L1 ratio in both ALS and FTD patients (32). Finally, whether chitinase levels also reflect the poorer outcomes associated with factors like bulbar onset or genetic status (e.g., C9orf72) needs further investigation.

## Chitinases Across the ALS-FTD Spectrum

Studies focusing on the broader ALS-FTD spectrum have noted that the two conditions present with specific chitinase dysregulation patterns. When examined alongside glial activation markers, these suggest different underlying inflammatory processes: increased microglial (as evidenced by CHIT1) and astroglial (as evidenced by CHI3L1) activation in ALS and FTD, respectively.

For instance, although CSF CHIT1 is elevated in FTD patients relative to both HCs and asymptomatic MCs, it is significantly higher in ALS patients (27, 33). Furthermore, CHIT1 immuno-staining in post-mortem spinal cord tissue was observed only in ALS cases, where it co-localized with IBA1-positive microglia and CD68-positive macrophages, and not in other neurodegenerative disorders, including FTD and AD (27, 33). Conversely, despite considerable overlap, CSF CHI3L1 levels were higher in patients with sporadic FTD relative to those with sporadic ALS, albeit only slightly. CHI3L1 elevation also correlated with cognitive dysfunction, as assessed by the Edinburgh Cognitive and Behavioral ALS Screen (ECAS), suggesting that it skews more closely to the FTD phenotype (28). Illán-Gala et al. (32) reported that although neither absolute CSF CHI3L1 levels nor the sAPPβ:CHI3L1 ratio significantly differed between FTD and ALS patients, CHI3L1 and global cognitive performance only correlated in the FTD subgroup. Furthermore, a robust inverse correlation was noted between the sAPPβ:CHI3L1 ratio and the FTD-Clinical Dementia Rating score in FTD patients. CHI3L1 immunoreactivity has been observed in astrocytes, but not microglia and neurons; its expression correlates with GFAP, particularly in acute inflammatory conditions like multiple sclerosis, suggesting that CHI3L1 is indicative of reactive astrocytosis (18, 19, 37). Crucially, negligible CHI3L1-positive astrocytes were observed in post-mortem ALS cortical tissue and no significant differences in *GFAP* mRNA in the spinal cord were noted between ALS patients and HCs (18, 35). CSF GFAP levels were also significantly increased in FTD patients while they were unaffected in ALS patients (27).

In summary, while the chitinases may not be specific markers for either condition, they allude to distinct neuroinflammatory profiles. If corroborated by other modalities, e.g., PET imaging (38), these profiles could help delineate the underlying pathology and provide specific targets for immunomodulatory therapy.

## Chitinases in the Broader Neuroinflammatory and Neurodegenerative Milieu

While much remains unknown about their cellular origin, it is evident that chitinase expression is not exclusive to ALS. It has been noted in multiple neurodegenerative conditions, where it predicts both clinical severity and long-term risk (39–41). The chitinases also robustly correlate with established neurodegenerative markers, including, e.g., the neurofilaments (10, 27, 31) and both total and phosphorylated tau (40, 42). Studies investigating multivariate panels have additionally reported close links to other inflammatory mediators. For instance, CSF chitinase levels correlated with MCP-1, and C-reactive protein in ALS patients and soluble TREM2 in cognitively unimpaired individuals (10, 28, 43). Transcriptomic studies have shown that *CHIT1* correlates with *IL-16, IL-18*, and *CHI3L1* and *CHI3L2* with complement *C1s* subcomponent (36, 41). Therefore, it is probable that the chitinases reflect the inflammation that is characteristic of the wider neurodegenerative process. Given the evidence from post-mortem co-localization studies and that significant dysregulations have been primarily observed in CSF rather than blood, we further speculate that the chitinases are proxies for reactive gliosis. It is worth noting, however, that systemic conditions may also influence chitinase levels, potentially “masking” alterations in blood.

While there is considerable overlap between neurodegenerative conditions, expression patterns differ, underscoring the different pathomechanisms at play; for instance, while CSF CHI3L1 increases as cognitive deficits worsen along the AD continuum, no similar associations have been noted with the ALSFRS-R, the primary indicator of disease severity in ALS (40). However, limitations with using the ALSFRS-R and derived parameters have been previously described (44). Instead, disease progression models could be particularly informative, as they allow interpretation of biomarker profiles within the disease course.

It is also imperative to expand beyond studying the chitinases as just fold changes within a case–control paradigm, given the evidence that they act as active immune modulators rather than just passive indicators of pathology. For instance, TNF-α, LPS, and IFN-γ stimulation increased both *CHIT1* expression and activity in human macrophages (45). Conversely, CHIT1, CHI3L1, and AMCase stimulation increased the transmigratory capacity of leukocytes from patients with multiple sclerosis (46).

In conclusion, studies should address how immune activation—vis-à-vis chitinase elevation—presents across the ALS disease course, whether it differs between glial cell types and what the functional consequences are. Studies also need to account for physiological aging, given multiple reports that it influences chitinase levels (27, 31, 47).

## Conclusions and Future Directions

What can be concluded of the chitinases holds true for all biomarkers; no single molecule can capture all the pathogenic processes at play in a disease as heterogeneous as ALS. This is particularly relevant in the case of inflammatory markers: these cannot be viewed in isolation because of their functional abundance and intricate signaling networks. It is the interaction with the disease microenvironment and the interplay between different cell types that drives pathology, rather than the singular action of a specific target. Multivariate biomarker panels are more likely to capture the dynamic immune signatures associated with different functional disease phases and identify optimal treatment windows and patients who would most benefit from immunomodulatory therapies. Therefore, the chitinases represent valuable additions to the current immuno-biomarker repertoire; while their diagnostic and prognostic efficacy is unlikely to supersede that of the neurofilaments, they can assist with subtle distinctions between different neurodegenerative conditions and delineate the mechanisms underlying glial dysregulation. Additional mechanistic studies could focus on how the chitinases reflect the dynamicity of glial cell responses across the disease. For instance, current evidence already indicates that the chitinases reflect a neuroinflammatory component that is common to both genetic and sporadic forms of ALS (27). Future prospective studies could focus on recruiting MCs and following them as they transition to clinical disease to better understand how chitinase elevation manifests, what triggers it, and how it relates to other modalities.

## Author Contributions

All authors listed have made a substantial, direct and intellectual contribution to the work, and approved it for publication.

## Conflict of Interest

The authors declare that the research was conducted in the absence of any commercial or financial relationships that could be construed as a potential conflict of interest.

## Abbreviations

AD: Alzheimer's Disease
ALS: Amyotrophic Lateral Sclerosis
ALSFRS-R: Amyotrophic Lateral Sclerosis Functional Rating Scale Revised
CHIT1: Chitotriosidase
CHI3L1: Chitinase 3-Like 1
CHI3L2: Chitinase 3-Like 2
CLs: Chi-lectins
fALS: Familial ALS
FTD: Frontotemporal Dementia
gALS: genetic ALS
GH18: family 18 Glycosyl Hydrolases
HCs: Healthy Controls
IFN-γ: Interferon gamma
IL-6: Interleukin 6
LMN: Lower Motor Neuron
LPS: Lipopolysaccharide
MCs: Mutation Carriers
MCP-1: Monocyte Chemoattractant Protein 1
MS: Mass Spectrometry
NDCs: Neurological Disease Controls
NfL: Neurofilament Light Chain
PLS: Primary Lateral Sclerosis
pNfH: Phosphorylated Neurofilament Heavy Chain
PR: Progression Rate
sALS: sporadic ALS
sAPPβ: soluble Amyloid Precursor Protein Beta
Th2: T Helper Type 2
TNF-α: Tumor Necrosis Factor alpha
TREM2: Triggering Receptor expressed on Myeloid Cells 2
UMN: Upper Motor Neuron.
